# Wellesley Female Institution

**Published:** 1831

**Authors:** 


### WELLESLEY FEMALE INSTITUTION.

REPORT OF THE WELLESLEY FEMALE INSTITUTION. By *Samuel Cusack*, M.B. Assistant Surgeon to the Institution for Diseases of Children.

[Dublin Hospital Reports, Vol. V.]

THE business of the above institution is conducted by a superintending accoucheur, assisted by competent pupils who reside in the establishment, recourse being had occasionally to consultants. The number of labour cases since the opening of the institution (the time not stated) up to the end of 1828, was 398, out of which, three cases only required instrumental aid —one the forceps, two the perforator. There were twelve cases of preternatural labour—four, where the upper extremities presented—eight, where the breech or feet presented. In all the four, where the upper extremities presented, turning was performed—and all the lives of the mothers were saved. In two, the children were still-born. . In one case, the contraction of the uterus was so violent, the hand was obliged to be withdrawn, and 120 drops of laudanum exhibited, after which the operation was performed with facility.

In presentations of the lower extremities the mothers were all saved, and the only instances where the infants were sacrificed were those where the head had been jammed in the pelvis by ill-directed attempts at extraction previously to admission. These cases were almost entirely left to themselves till the breech was expelled, when the usual attention was paid to insure that the face of the child should be turned towards the sacrum of the mother. After the extraction of the arms, the chin was depressed by placing the finger in the mouth of the child in the usual way, so as to give the head the direction of the axis of the pelvis, and to cause the biparietal, instead of the occipito-mental, to be the moving diameter.

"In five cases the funis was protruded. In some of these cases the pulsation had ceased previous to application for assistance; in the others none of the means recommended in such cases for the preservation of the child appeared admissible. Of six cases of puerperal convulsions, two occurred between the fifth and eighth month; in one the fits were always induced by constipation of the bowels, and after this cause was removed, did not re-appear. One case occurred after parturition; the cause was similar to that of the preceding case. The patient recovered under the employment of venesection, and purgatives; of the remaining cases of convulsions, all of which occurred during labour, one patient was delivered by turning, during which operation the fits were suspended, but recommenced after delivery, and carried the patient off. Another was delivered by the crotchet, who recovered from the convulsions, but subsequently died of peritonitis. In another case (a first pregnancy) the convulsions appeared at the commencement of labour, the membranes were ruptured by the finger, twenty ounces of blood taken from the temporal artery, cold applied to the head, and injections and purgatives administered. The fits however continuing, the forceps were applied when the head was sufficiently low, and mother and child were both saved.

The convulsions appeared in one instance about twelve hours after the birth of the first twin, the woman having been improperly allowed to remain undelivered of the second all that time. The forceps were promptly applied, and the second child extracted without difficulty, but dead. In this case, instead of the patient being comatose between the fits, she exhibited all the symptoms of delirium ferox, the birth of the second child not seeming to have any effect on her condition, but after the extraction of the placenta she became perfectly tranquil, and the fits did not again appear." 498.

In a recent case which the author attended with Dr. Nicholson, and where eight fits of well-marked convulsions occurred in the course of twelve hours, the os uteri was found dilated to the size of a half-crown, the head presenting, and the membranes ruptured. By means of copious bleeding, shaving the head, and cold applications, with the internal use of scammony and calomel, and lavements, the convulsions were completely subdued, and the patient was delivered naturally of a dead child, after an interval of thirty hours, during which she remained rational.

"This case contrasted with one already related, where the convulsions had ceased on the delivery of the patient by the perforator, but in which fatal peritonitis supervened, would lead us to conclude, that artificial delivery ought to be limited, except when the pelvis is deformed, to those cases where the forceps can be used, and that turning, or the perforator, should be employed only in those cases where, from the condition of the parts, there appears no risk of exciting inflammation: indeed unless there be strong proof of the death of the child, or we have to deal with a narrow pelvis, it does not seem that under any circumstances is the use of the perforator justifiable. There can be no doubt that convulsions will often cease on artificial delivery being performed, even though in a rude violent manner, but the result in such cases usually is the death of the patient by peritonitis." 500.

The author considers that opening the bowels is not of less consequence in the treatment of puerperal convulsions, than bleeding. Cold to the head is a very powerful auxiliary.

There are cases, he believes, where the disease is the result of nervous irritability rather than of plethora, and, in such cases, the too free abstraction of blood will only hurry the disease on to a fatal termination.

Hæmorrhage occurred in six cases during labour, caused by the attachment of a small portion of placenta over the os uteri. In all, it was arrested by the rupture of the membranes. In fifteen cases it was deemed necessary to remove the placenta by the hand. The number is large but is accounted for by the fact that many of them were cases where assistance was called for on account of retention of the placenta alone, the consequence of previous mismanagement. In five out of these fifteen cases, there was hour-glass contraction of the uterus. In one only was there any difficulty in the removal of the placenta, where, co-existent with the stricture of the uterus, there was violent uterine action, and high excitement, both nervous and vascular. Venesection and opiates were employed previous to any attempts at extraction. These, however, did not lessen the difficulty of the operation, and the patient died of venous hæmorrhage ten minutes afterwards.

"In five cases hemorrhage occurred previous to the delivery of the placenta. Two of these cases were of hour-glass retention: in one sudden death took place about six hours after delivery, although the placenta had been removed without difficulty, and the patient appeared to have completely recovered from the loss of blood, which had not been at all extensive; no hemorrhage occurred externally, nor on the post mortem examination did any appearance present itself sufficient to account for her death. The uterus had contracted well, and no coagula were found in its cavity." 502.

A few hæmorrhages after the delivery of the placenta were arrested by pressure over the uterus, the application of cold, quietude, and fresh air.

"A considerable number of abdominal inflammations presented themselves; at particular periods they were exceedingly prevalent; at other times equally rare. The type of these inflammations varied with the periods of their appearance. In December and January, 1827-28, the peritoneum seemed to be the structure most deeply engaged, and the inflammation to be of a phlegmonous character. In March the disease assumed the low typhoid character. In May, 1828, several cases were met with in which the intestinal mucous membrane was the seat of disease; they were characterized by thirst, redness of tongue, or white coating with florid papillæ interspersed, intolerance of light, headache, and obscure abdominal tenderness." 503.

Cases of abortion were very numerous. Relief was sought, in some cases, for hæmorrhage, in some, for retention of part of the ovum—in others, for derangement of general health consequent on the miscarriage. Hæmorrhages were easily suppressed by the usual means. Excepting after the sixth month, manual extraction of the placenta was not attempted. Enemata, purgatives, friction of the abdomen, and binding were the means employed for expulsion.

"Amongst the most frequent of the diseases of females, were those connected with the function of menstruation. In the treatment of these cases more attention was paid (with some exceptions) to the constitutional, than to the local symptoms, and what are considered specific or directly emmenagogue medicines were but rarely exhibited, and never found effectual. The catamenial derangements consisted in total suppression, in diminution, in excess, in irregularities attendant on their final cessation, and in distressing accompanying symptoms.

These states were accompanied by two very opposite conditions of the system, debility and plethora. In the former, the object principally held in view was to improve, as much as possible, the general condition of the system; in the latter and less frequent condition, depletion, either topical or general, was employed." 504.

Cancer uteri was often met with. In all instances, the disease was so extensive as to engage all the soft parts in the neighbourhood of the os uteri:—thus shewing that extirpation of the uterus, at that period of the disease, was totally inapplicable.

"Some instances occurred in which the os uteri was tumefied, irregular, and tender to the touch, accompanied by a muco-sanguineous discharge, by pain about the back and thighs, anasarca of the lower extremities, loss of appetite, debility, and sallowness of the countenance. They were treated with alterative doses of the pil. hydrargyri, followed by mild saline purgatives combined with bitters; strict attention was paid to their general and dietetic management, and in every instance a perfect, though in some a gradual, recovery ensued." 505.

The above is an important fact, and points out the necessity of strict investigation, before we condemn, as malignant, cases that may be only obstinate or tedious—and thus submit patients to the hazard of a dangerous, often an unsuccessful operation.

Two cases of polypus uteri occurred, in which the patients were reduced to a state of great debility. The tumours were removed by the ligature, and the patients recovered perfectly.

"In one case of polypus uteri, where the patient had been exceedingly debilitated, the pulsation of the large vessels about the neck was visible at a distance for some months, so that, on a superficial inspection, she might have been supposed to labour under disease of the heart." 506.

Mammary abscess was frequently arrested in its progress by the exhibition of emetic tartar. The secale cornutum was employed in 12 or 14 cases. In six it produced no sensible effect; but there was reason to believe that the medicine was not good. In three instances, where the dose was half a drachm at a time, apoplectic symptoms ensued.

"In a case of breech presentation, in a female who had borne several children, ten grains of ergot, given in infusion, were administered; she had not had any pains for the entire of the preceding night. Pains, however, came on so immediately after the administration of the ergot, as to leave no doubt on the author's mind of its efficacy in that instance. Amongst other instances, a case was treated by Mr. Dashwood, an extremely intelligent pupil, where the placenta, after three hours' retention, was expelled by uterine action consequent on the administration of the ergot, though in the two preceding deliveries of the same patient the placenta was extracted by the hand." 508.

One case of severe puerperal inflammation of the joints occurred. The placenta had been removed in consequence of hæmorrhage, and the patient was going on favourably till the 7th day, when she was attacked with febrile symptoms and inflammation of the knee and ankle of one leg. The fever was of a mixed character, accompanied by much gastric derangement and acceleration of the pulse, without hardness. The pain in the parts preceded the other symptoms of inflammation; but, in a short time, the joints became red and swollen, the calf of the leg participating in the tumefaction. The pain was so violent as to deprive the patient of rest, and to require large opiates. Calomel, opium, and emetic tartar to ptyalism constituted the principal means of cure, with frequent application of leeches. Sulphate of quinine, with compound tincture of gentian was found useful towards the close of the malady. "*In no stage whatever of the disease were any symptoms of venous inflammation discernible.*"

"Two cases of hydatids of the uterus were treated; both of the individuals were married, and one had previously children, and experienced, with the exception of feeling the movements of the child, the usual symptoms of pregnancy. One patient had a constant discharge of a yellowish colour; the other was free from any vaginal discharge till a few days before the expulsion of the hydatids, when there was a slight discharge of blood. In one instance the hydatids were expelled without much accompanying hemorrhage; in the other, there was a considerable loss of blood. The patients were treated, after the expulsion of the hydatids, like puerperal patients; one of them had a considerable quantity of milk in the breasts for a few days, and has since borne a living child. The hydatids expelled in one case amounted to upwards of a gallon; they were of an elliptical elongated shape, connected together by delicate pedicles, and surrounded by a cyst, resembling the decidua." 512.

One patient had a malignant tumour of the os uteri. Five months previous to application she was in good health. She had borne several children.

"On examination per vaginam, a tumor, as large in circumference as a dollar, but much thicker, was found growing from the lower part of the cervix uteri. It was firm and elastic, and a portion of the cervix uteri could be felt above the tumor, apparently free from disease. After endeavouring as far as it was possible to improve the patient's general health, a ligature was applied as high up as the cervix uteri, by means of the common double canula." 513.

In the progress of the case the ligature was tightened occasionally; but, on the 16th day, the cervix uteri, not being completely divided, was drawn down, and severed across with a bistoury. For upwards of a month, the patient appeared to be doing well; recently, however, the ulceration recommenced at the place where the tumour was separated, and all the former distressing symptoms have returned. Still the case is important, as shewing how far the uterus will bear, with impunity, the application of the ligature, there being no threatening of peritoneal inflammation or retention of urine, during the time the ligature was on the uterus. The author, in future, would prefer the knife to the ligature. We must close this review of Dr. Cusack's paper with the following case.

### Uterine Hæmorrhage after Delivery.

"The patient, a healthy young woman, was much excited, apparently in consequence of having taken some spirits. Her face was much flushed, pulse 130, full and strong; enemata and aperient medicines had been administered without any effect, and no uterine action existed. As the vascular excitement seemed the result of a temporary cause, it was not deemed necessary to have recourse to venesection; and lest some untoward event should occur, it was deemed advisable to deliver the patient; accordingly turning was performed, not without some difficulty, in consequence of the height in the uterus at which the child was placed. It however was born alive, and what was remarkable, very soon became a fine child, while the infant that was born naturally died in a few days.

Every means were taken to promote the safe expulsion of the placenta, which in about half an hour was expelled naturally, and the uterus became hard and well contracted. In a short time however, most violent uterine hæmorrhage came on; cold was promptly applied to the region of the uterus, and pressure made over that viscus, by which means the hemorrhage soon ceased.

In this instance the patient did not become at all faint, nor was any internal stimulus employed except cold water, and the only effect produced by the loss of blood was the reduction of the pulse from 130 to 90, which also became proportionally soft; a very desirable result. This was evidently a case of hemorrhage resulting from vascular excitement, and shews the necessity of preventing, by attention to temperature, diet, drinks, &c. during labour, so unfavourable a condition of the circulation." 517.

